# Can ratio of blood/curettage material HCG be used as a reliable method for differentiating miscarriage and ectopic pregnancy?

**DOI:** 10.12669/pjms.295.3391

**Published:** 2013

**Authors:** Gokhan Acmaz, Huseyin Aksoy, Evrim Bayraktar, Muruvvet Baser, Gokmen Zararsiz, Iptisam Ipek Muderris

**Affiliations:** 1 Gokhan Acmaz, MD, Assistant Professor, Dept. of Obstetrics and Gynecology, Kayseri Training and Education Hospital of Medicine, Kayseri, Turkey.; 2 Huseyin Aksoy, MD, Dept. of Obstetrics and Gynecology, Kayseri Military Hospital Kayseri, Turkey.; 3 Evrim Bayraktar, PhD, Assistant Professor, Dept. of Gynecology and Obstetric Nursing, Erciyes University Faculty of Medicine, Kayseri, Turkey; 4 Muruvvet Baser, PhD, RN. (Head of Department), Associate Professor**, **Dept. of Gynecology and Obstetric Nursing, Erciyes University Faculty of Medicine, Kayseri, Turkey; 5 Gokmen Zararsiz, Department of Biostatistics, Erciyes University Faculty of Medicine, Kayseri, Turkey; 6 Dr. Iptisam Ipek Muderris, Professor and Head, Dept. of Obstetrics and Gynecology, Erciyes University Faculty of Medicine, Kayseri, Turkey

**Keywords:** Miscarriage, Ectopic pregnancy, Beta-hCG, Curettage

## Abstract

***Objective*** **: **There is an increasing rate of ectopic pregnancy with the use of assisted reproductive techniques. There is currently no serum test to differentiate ectopic pregnancy from miscarriage. Early and accurate detection of ectopic pregnancy may prevent the development of complications. The aim of this study was whether the ratio of serum/curettage material hCG could provide us with reliable and early diagnosis in distinguishing miscarriage and ectopic pregnancies and also to measure the diagnostic accuracy rate of this method.

***Methods:*** A total of 24 patients were evaluated. Twelve of them were diagnosed as an ectopic pregnancy and 12 of them were diagnosed as a miscarriage. All the patients’ diagnoses were confirmed pathologically. Non-of the patient had viable fetus. All participants underwent curettage protocol. Serum and curettage material were obtained at the same time. HCG measurements were done from blood and curettage material.

***Results:*** Ratio of Blood/Curettage Material HCG provides fast and reliable results within a few hours with 91.7% accuracy rates.

***Conclusion:*** Ratio of blood/curettage material HCG can be used as a reliable method for differentiating ectopic pregnancy and miscarriage.

## INTRODUCTION

Ectopic pregnancy (EP) refers to the implantation of a viable ovum outside the uterine corpus, which is a significant cause of maternal morbidity and mortality. There is no single test for its early diagnosis and current diagnosis relies on serial hCG levels and ultrasound examination when the location of the pregnancy is unclear on initial presentation.^1^ Early and accurate detection of EP may prevent the development of complications such as tubal rupture, intra-abdominal hemorrhage and may allow for a medical treatment.^2^

The diagnosis of EP should not be based on an inability to visualize a normal intrauterine pregnancy (NIP), but rather on the positive visualization of an adnexial mass using transvaginal sonography (TVS). In 8–31% of women who are admitted to a clinic for early pregnancy (hCG>5), sac may not be visualized by TVS. These women are classified as having a “pregnancy of unknown location” (PUL).^3^^,^^4^ In rare cases there is no evidence of trophoblastic tissue on ultrasound or at laparoscopy and the serum hCG levels are low and have reached a plateau (<500U/l). These have been classified as persisting PUL.^5^ Two of the most common symptoms that overlap in both EP and abortion are vaginal bleeding and pelvic or abdominal pain.^6^^,^^7^

Nevertheless, 23–29% of PUL require surgical intervention due to a worsening clinical condition or non-declining serum hCG, and with experience, intervention rates can be as low as 9%.^8^^,^^9^

The concept of combining ultrasound with measurements of serum hCG using a discriminatory zone is well described. Barnhart et al. showed that above a discriminatory level of 1500IU/l an intrauterine gestation sac was seen in 91.5% of cases.^10^ In women without an ectopic mass or fluid in the pouch of Douglas, Mol et al. used a serum hCG cut-off of at least 2000IU/l.^11^ In NIP there should be a 66% rise over the baseline value over 48 hour.^12^ Approximately 13% of EP and 15% of NIP screened in this way appear to be abnormal, giving contradictory results and delaying the diagnosis beyond 48hour.^13^

Curettage protocol is used for the management of ectopic pregnancy and abnormal uterine pregnancies in many clinics. The aim of this study was to determine whether the hCG ratio of blood serum versus curettage material could provide an early diagnosis, which would improve the prognosis of EP. In other words, this ratio may be helpful for instantly discriminating early EP from miscarriages.

## METHODS

***Participants and Procedures: ***This study was conducted at the Kayseri Education and Training Hospital of Medicine from May to December 2012. We prospectively studied 26 consecutive patients with miscarriages and EP presenting with positive β-hCG, abdominal pain, vaginal bleeding and history of amenorrhea who were admitted to our tertiary centre emergency clinic.

Patients who were admitted in our clinic with positive β-hCG, abdominal pain; vaginal bleeding and history of amenorrhea were investigated. We began to follow protocol and serial β-hCG measurements. Patients’ blood hCGs were measured with an interval of two days and HCG test was performed on each patient at least 3 times. Before dilatation and curettage procedure, serial β-hCG levels of all patients declined, rising abnormally or having plateau during follow up protocol. Therefore, possibility of viable pregnancies was eliminated. After serial hCG follow up protocol, all patients underwent curettage protocol. Blood and curettage material samples were obtained at the same time. Pregnant women who have viable fetus was our exclusion criteria. The patients with hCG secreting tumors and gestational trophoblastic diseases were excluded from the study, as well. We selected ectopic pregnancies in those who required surgical intervention for EP and confirmed this situation with hisyopathological examination. On the other hand miscarriages were diagnosed with presence of chorionic villi. 

Patients were matched for gestational age based on last menstrual period, ethnicity, maternal age, gravida, parity and other clinical parameters. EP and miscarriage were diagnosed based on vaginal examinations, serial β-hCG levels, USG findings, hystopathological examination of curettage material and laparoscopic evaluation at first and finally by hystopathological examination. If the clinician was unable to make a diagnosis on the first visit, the patient was followed until a diagnosis was confirmed. All cases had histological confirmation of the diagnoses. All patients included in the EP group were managed by salpingectomy or salpingostomy and histological diagnosis of tubal pregnancy was made on all excised specimens.

Patients were separated into two groups according to their diagnoses. Group 1 (*n*= 12) was constituted from EPs and group 2 (*n*= 12) was constituted from miscarriages. One case in miscarriage group because of data loss and one case in EP group because of insufficient curettage material were excluded from the study. 

All blood samples were collected before treatment by peripheral venous puncture as soon as patients arrived with the presenting symptoms as stated above. Patients’ blood hCGs were measured with an interval of two days and HCG test was performed on each patient at least 3 times. Before dilatation and curettage procedure, serial β-hCG levels of all patients declined, rising abnormally or having plateau during follow up protocol. Therefore, possibility of viable pregnancies was eliminated. After serial hCG follow up protocol, all patients underwent curettage protocol. Blood and curettage material samples were obtained at the same time.

Dilatation and curettage procedures were performed under conscious sedation. Final diagnosis was based on the presence or absence of chorionic villi on permanent histology of uterine curettage.^14^ When salpigostomy or salpingectomy was performed, pathological analysis of the removed tissue confirmed the diagnosis of PE. Sonography (Hitachi EUB 415/515; Hitachi Medical Corporation, Tokyo/Japan) was performed by one of the study investigators.

After blood and curettage material were obtained synchronously during curettage protocol, both the materials were centrifuged with 4000 rpm. Supernatant of curettage material was separated for hCG measurement and sediment of that was separated for pathologic examination. The both blood and curettage material hCG concentration was determined with the use of chemiluminescens method. (hCG kit: immulate 2000 xpico359 Lranberris Gwynedd LL55UK precision 4.8%, analyzator: Siemens Healthcare Diagnostics Product Ltd. Flanders/USA) and expressed in international units per liter. Then ratio of blood/curettage material hCG was calculated and results were correlated with pathologic examination.

This study was approved by the Ethics Committee of Erciyes University hospital ethical review board and was designed in accordance with the Declaration of Helsinki. All patients entered this study only after informed consent was obtained.

***Power:*** To determine the sample size we performed power analysis and made a pilot study for this. The blood and IU HCG values of five ectopic pregnancies and three abortions who performed at Kayseri Training and Education Hospital throughout one moth were taken into study. Using the blood HCG / IU HCG ratios of these patients as base, a power of 95%, an alpha of 0.05, and the unpaired *t *test, we calculated that 10 patients were enough in each group. PASS 11 software (NCSS, Kaysville, UT, USA) was used for performing these analyses. Because of expected dropouts from the study (such as data loss and ineligibility for inclusion), we decided to include 26 patients. Then we were capable of studying on 24 patients.

***Statistics: ***To check the normality of data, Shapiro Wilk’s test was used and histograms, q-q plots were examined. Independent-samples *t* test and Mann-Whitney U tests were used due to the normality results of variables and values were expressed as mean ± standard deviation or median, 25^th^ and 75^th^ percentiles. Receiver operating characteristic (ROC) curve was drawn for blood HCG/curettage material HCG (iu hCG) value in order to identify EP and miscarriage, and the area under ROC curve value was calculated with 95% confidence interval (CI). Also, statistical diagnostic measures and Kappa test results were given with 95% CIs for the determined cut-off value. MedCalc (Version 9.2.0.1) software was used for analyses with considering a *p*<0.05 probability level statistically significant. 

## RESULTS

There was no difference between groups for gravity and parity. Age and ratio of blood/curettage material HCG were significantly high in EP group. Comparison of both groups for age, gravity, parity and blood/curettage material HCG is shown in Table-I.

**Table-I:** Comparison of both groups for age, gravity, parity and blood/curettage HCG

*Features*	*Abortion*	*Ectopic Pregnancy*	*p*
Age (years)	26.8 ± 6.6	32.8 ± 6.4	0.035
Gravida	2.0 (2.0-3.5)	3.5 (2.0-4.0)	0.443
Parity	1.0 (0.0-1.5)	2.0 (0.5-2.0)	0.143
Blood/curettage HCG	0.1 (0.1-0.4)	2.7 (1.4-4.6)	<0.001

**Values are expressed as mean ± SD or median (25** ^th^ **/75** ^th^ **percentiles)**

**Fig.1:**
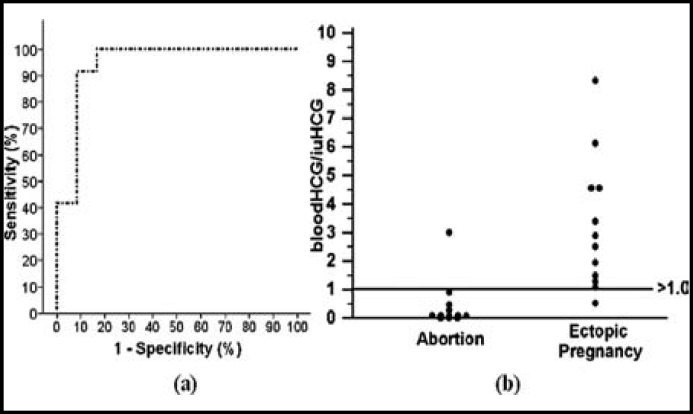
The ROC curve and distribution of blood/curettage material HCG values in EP and abortion. (a) The Receiver operating characteristic (ROC) curve of blood HCG/ curettage HCG in order to identify EP and abortion The area under the ROC curve value was 0.94 (0.85-0.99) and significant (*p*<0.001). (b) The distribution of blood HCG/curettage material HCG values in abortion and ectopic pregnancy group around the value of 1.

The ROC curve and distribution of blood/curettage material HCG values in EP and abortion is shown in Fig.1.

Sensitivity, specificity, positive and negative predictive rates, accuracy rate and kappa statistics of this new method are shown in Table-II.

**Table-II:** Statistical diagnostic measures of blood HCG/curettage material HCG for identification of EP and abortion

*Diagnostic Measure*	*Value (95% CI)*
Sensitivity	91.7 (61.5-99.8)
Specificity	91.7 (61.5-99.8)
Positive Predictive Rate	91.7 (61.5-99.8)
Negative Predictive Rate	91.7 (61.5-99.8)
Accuracy Rate	91.7 (73.0-99.0)
Kappa statistic	83.3 (61.2-99.9)^*^

Values are defined as percentages. ^*^p<0.001

## DISCUSSION

A large retrospective study of viable pregnancies showed that the median slope for the rise of serum beta-hCG was 2.24 after 2 days (or a 124% rise).^15^ Thus two serum beta-hCG levels measured 48h apart can help the diagnosis of EP. A 66% rise in beta-hCG level was highly suggestive of a NIP, although 15% of such cases still turned out to be EP. A rise in the beta-hCG concentration of <66% made the presence of an EP more likely (likelihood ratio of 25). A fall in the beta-hCG by less than 50% was almost always associated with an abnormal pregnancy, while a fall of more than 50% was highly suggestive of a spontaneous miscarriage. Case cohort studies showing a decline in the beta-hCG concentration of >50% ruled out the possibility of an EP and a NIP (likelihood ratio 0).^16^

Although this algorithm is used widely in clinics, only measurement of hCG will not be diagnostic in the majority of cases. When the serum hCG is above the discriminatory zone, in most cases it will be large enough to be visualized by ultrasonography. Problems arise at lower serum hCG levels or in the smaller number of cases when an ultrasound diagnosis cannot be made. In such cases it is possible to distinguish between a PUL which will develop into a NIP and those that subsequently become EP on the basis of serum hCG increase over 48 hour.^12^

The aim of this study was to investigate whether ratio of blood hCG/curettage material hCG could be used as a reliable and fast diagnosis method for differentiating miscarriage and EP in early state. According to our knowledge, this method has not been described previously. We hypothesized that if blood hCG/curettage material hCG is greater than one, the diagnosis should be EP but if this ratio is less than one, the diagnosis should be miscarriage. The results have supported our hypothesis and this method may help clinicians to diagnose EP and miscarriage in early state. Early diagnosis of EP allows fertility preserving treatment or prevents patients from serious complications of EP. Our results show that accuracy rate of this method is 91.7% and this new method provides results within a few hours.

It is well known that human trophoblast differentiates into two pathways: extravillous cytotrophoblasts (EVCT) that invade the uterus wall and villous cytotrophoblasts (VCT) that fuse to form the syncytiotrophoblast (ST) involved in placental exchanges and endocrine function. Then Handschuh K et al^17^ compared hCG secretion by primary cultures of VCT and EVCT isolated from the same first trimester human chorionic villi. They proved that invasive EVCT also expressed and secreted high levels of hCG, suggesting a specific paracrine and/or autocrine role for hCG from EVCT origin.^17^ One way to interpret the present findings is to conclude that invaded by EVCT, uterus wall can be accepted as a rich source of hCG in NIP and this finding cannot be found in EP.

Some of the authors have proved that hCG, in early pregnancy, is found in coelomic fluid in higher levels than in maternal circulation.^18^^,^^19^ This data could be interpreted that we can find higher intra-uterine hCG levels than blood in abortion. This method allows fast reliable diagnosis and allows discrimination of the EP and miscarriage in early state. Although the advantages of this method are described above, there are some disadvantages, as well. 

If complete spontaneous losses of an intrauterine pregnancy (CSLIP) occur, cytotrophoblasts cannot be found in uterus; therefore, blood hCG levels may be higher than intra-uterine hCG in miscarriage. On the other hand, cervical ectopic pregnancy can be diagnosed as an abortion with this method but this hypothesis has validity for pathologic examination, too. If TVS yields a non-diagnostic result with no direct evidence of a NIP or EP, histology of endometrial curetting showing chorionic villi will exclude an EP. However, absence of chorionic villi does not always confirm an EP, since chorionic villi will also be absent in patients with CSLIP.^20^

Ratio of blood hCG/curettage material hCG can be considered as a new diagnostic approach and it provides fast, early and reliable discrimination of EP and miscarriage in early state. Detection and discrimination of EP and abortion easily **may** be made by using this method when TVS yields non-diagnostic result. Our study group, however, is relatively small and this study requires large scale study groups with other markers.

## Notes

***Note:*** Kayseri Training and Education Hospital financially supported this study.

## References

[1] Horne AW, Shaw JL, Murdoch A (2011). Placental growth factor: a promising diagnostic biomarker for tubal ectopic pregnancy. J Clin Endocrinol Metab.

[2] Nadukhovskaya L, Dart R (2001). Emergency Management of the Nonviable Intrauterine Pregnancy. Am J Emerg Med..

[3] Hahlin M, Thorburn J, Bryman I (1995). The expectant management of early pregnancies of uncertain site. Hum Reprod.

[4] Banerjee S, Aslam N, Zosmer N (1999). The expectant management of women with pregnancies of unknown location. Ultrasound Obstet Gynecol.

[5] Condous G, Okaro E, Bourne T (2004). The management of ectopic pregnancies and pregnancies of unknown location. Gynecol Surg.

[6] Gracia CR, Sammel MD, Chittams J (2005). Risk factors for spontaneous abortion in early symptomatic first trimester pregnancies. Obstet Gynecol.

[7] Della Giustina (2003). Ectopic pregnancy. Emerg Med Clin North Am.

[8] Condous G, Okaro E, Khalid A (2003). Should complete miscarriages be followed up with serum human chorionic gonadotrophin levels?. Ultrasound Obstet Gynecol..

[9] Banerjee S, Aslam N, Woelfler B (2001). Expectant management of early pregnancies of unknown location: a prospective evaluation of methods to predict spontaneous resolution of pregnancy. Br J Obstet Gynaecol.

[10] Barnhart KT, Simhan H, Kamelle SA (1999). Diagnostic accuracy of ultrasound above and below the beta-hCG discriminatory zone. Obstet Gynecol.

[11] Mol BW, Van der Veen F (2000). Results of expectant management of women early pregnancy and unknown location. Ultrasound Obstet Gynecol.

[12] Kadar N, Caldwell BV, Romero R (1981). A method of screening for ectopic pregnancy and its indications. Obstet Gynecol.

[13] Ling Stovall (1994). Update on the diagnosis and management of ectopic pregnancy. Adv Obstet Gynecol.

[14] http://www.ztb.gov.tr/kose-yazisi/dis-gebelik-ve-tedavi-secenekleri/12.

[15] Barnhart KT, Sammel MD, Rinaudo PF (2004). Symptomatic patients with an early viable intrauterine pregnancy: HCG curves redefined. Obstet Gynecol.

[16] Mol BW, Hajenius PJ, Engelsbel S (1998). Serum human chorionic gonadotropin measurement in the diagnosis of ectopic pregnancy when transvaginal sonography is inconclusive. Fertil Steril.

[17] Handschuh K, Guibourdenche J, Tsatsaris V (2007). Human chorionic gonadotropin expression in human trophoblasts from early placenta: comparative study between villous and extravillous trophoblastic cells. Placenta..

[18] Wathen NC, Cass PL, Kitau MJ (1991). Human chorionic gonadotrophin and cz-fetoprotein levels in matched samples of amniotic fluid, extraembryonic coelomic fluid, and maternal serum in the first trimester of pregnancy. Pren Diag.

[19] Jauniaux E, Gulbis B, Jurkovic D (1993). Protein and steroid levels in embryonic cavities in early human pregnancy. Hum. Reprod.

[20] Nama V, Manyonda I (2009). Tubal ectopic pregnancy: diagnosis and management Arch Gynecol Obstet.

